# The Repertoire Dissimilarity Index as a method to compare lymphocyte receptor repertoires

**DOI:** 10.1186/s12859-017-1556-5

**Published:** 2017-03-07

**Authors:** Christopher R. Bolen, Florian Rubelt, Jason A. Vander Heiden, Mark M. Davis

**Affiliations:** 1 Department of Microbiology and Immunology, Stanford University School of Medicine, Stanford, 94305 CA USA; 2 Genentech, Inc., 1 DNA Way, MS 93, South San Francisco, 94080 CA USA; 3 Interdepartmental Program in Computational Biology and Bioinformatics, Department of Computational Biology & Bioinformatics, Yale University, New Haven, 06520 CT USA; 4 Howard Hughes Medical Institute, Stanford University School of Medicine, Stanford, 94305 CA USA; 5 Institute of Immunity, Department of Microbiology and Immunology, Transplantation and Infection, Stanford University School of Medicine, Stanford, 94305 CA USA

**Keywords:** Repertoire sequencing, Immunology, Nonparametric methods

## Abstract

**Background:**

The B and T cells of the human adaptive immune system leverage a highly diverse repertoire of antigen-specific receptors to protect the human body from pathogens. The sequencing and analysis of immune repertoires is emerging as an important tool to understand immune responses, whether beneficial or harmful (in the case of autoimmunity). However, methods for studying these repertoires, and for directly comparing different immune repertoires, are lacking.

**Results:**

In this paper, we present a non-parametric method for directly comparing sequencing repertoires, with the goal of rigorously quantifying differences in V, D, and J gene segment utilization. This method, referred to as the Repertoire Dissimilarity Index (RDI), uses a bootstrapped subsampling approach to account for variance in sequencing depth, and, coupled with a data simulation approach, allows for direct quantification of the average variation between repertoires. We use the RDI method to recapitulate known differences in the formation of the CD4^+^ and CD8^+^ T cell repertoires, and further show that antigen-driven activation of naïve CD8^+^ T cells is more selective than in the CD4^+^ repertoire, resulting in a more specialized CD8^+^ memory repertoire.

**Conclusions:**

We prove that the RDI method is an accurate and versatile method for comparisons of immune repertoires. The RDI method has been implemented as an R package, and is available for download through Bitbucket.

**Electronic supplementary material:**

The online version of this article (doi:10.1186/s12859-017-1556-5) contains supplementary material, which is available to authorized users.

## Background

The B and T cells of the immune system of higher organisms create and express a vast array of different immunoglobulin (Ig) and T cell receptor (TCR) sequences, respectively, in order to target invading pathogens. During early stages of the cell maturation process, a set of V (variable), D (diversity) and J (joining) gene segments are chosen from a genetically encoded pool to create a typically unique receptor for each B and T cell, a process known as V(D)J recombination [1–3]. Recent studies have used deep sequencing combined with sophisticated computational pipelines to study the contents of these repertoires [4, 5], and repertoire datasets have begun to proliferate in publicly available sequencing databases like SRA, ImmPort [6], and VDJServer (https://vdjserver.org). However, although some groups have developed standardized terms for reporting and recording V(D)J analysis results like VDJML [7], there are relatively few widely available tools for analyzing data of this type.

A common task in the analysis of immune repertoire datasets is to examine the variation, or diversity, within an individual’s immune system. A number of metrics, including Shannon diversity, species richness, Simpson index, and the generalized Hill diversity, have been previously used as methods for estimating diversity or quantifying the level of clonal expansion [8]. Resampling strategies have also been used in order to compare diversity between sets of repertoires [9, 10]. Together, these methods have proved useful for understanding clonality, and for estimating and comparing the amount of clonal expansion between individuals’ repertoires. However, because individuals very rarely share overlap of specific rearrangements or specific receptor sequences, there have been few studies which directly compare the contents of B or T cell repertoires. In this paper, we present a tool for directly comparing sequencing repertoires, with the goal of quantifying the average difference in V, D, and J gene segment utilization between repertoires.

The problem of comparing the contents of a repertoire is not unique to studies of the immune system. Methods for comparing prevalence of individual bacterial species have been widely used in the field of metagenomics [11–13], and well-established parametric methods for comparison of individual genes have long been used in RNA-Seq experiments [14, 15]. However, these methods focus on comparison of individual species within a dataset, whereas direct sample-to-sample comparisons (e.g. correlation, Euclidean distance, etc.) rely on simple data transformations for normalization or subsetting to the most common species to remove bias from variation in sequencing depth. While such approaches are generally reliable for high-depth sequencing experiments, their performance will suffer as the effects of random sampling become more pronounced.

In our previous paper [4], we demonstrated the power and utility of quantifying immune repertoires via deep sequencing of sorted B and T cell populations. We used the Repertoire Dissimilarity Index (RDI) metric to compare V, D, and J gene usage within the naïve and memory repertoires of identical twins, and we showed that the contents of naive immune repertoires are determined primarily by heritable factors, and that observed biases in gene usage are carried over into the memory repertoire. Here, we describe and extend the RDI, a non-parametric, computational approach for the estimation of repertoire differences. We show that the distance metric is an accurate approximation of the true difference between two repertoires, and that this method accounts for the various challenges associated with repertoire profiling, namely varying sequencing depth and gene prevalence. The code for calculating the RDI metric has been implemented as an R package, and is available for download at http://bitbucket.org/cbolen1/rdicore.

## Methods

**Fig. 1:**
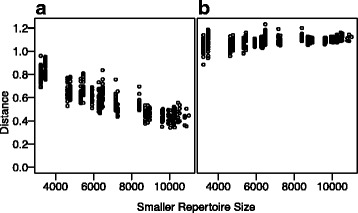
Repertoire subsampling accurately controls for variance inflation. A simulated sequencing dataset was generated by drawing 30 replicate samples from a single pool containing 50 genes of varying prevalence. For each replicate, the number of sequences was chosen randomly, and the total count varied between 3000 and 12,000. **a** The frequency of each gene was tallied, and the euclidean distance between each pair of replicates was calculated. **b** Each repertoire was subsampled to the size of the smallest repertoire (*n* = 3216), and euclidean distance was calculated based on normalized gene frequency in the subsampled dataset. The distance measurement was then averaged across multiple subsampling steps. All distance metrics are compared against the original repertoire size for the smaller repertoire

There are a number of challenges associated with direct comparisons of gene segment prevalence among immune repertoires. First, variations in genotype and copy number of individual V, D, and J gene segments leads to high variability in segment prevalence, often resulting in missing gene segments and orders of magnitude differences in frequencies. In addition, the variable sequencing depth in individual samples can result in higher variance in repertoires containing small numbers of sequences, leading to increased error in the estimates of segment prevalence—and therefore inter-repertoire distance—as the number of sequences in a repertoire decreases (Fig. 1a).

In order to account for these challenges and to make meaningful comparisons between repertoires of interest, we developed a multi-step process, the Repertoire Dissimilarity Index (RDI), which controls for variance inflation by calculating distance after subsampling all repertoires to the same size. In addition, we use a novel simulation approach to directly quantify the average difference between elements of the repertoire. We demonstrate the utility of this method both for identifying repertoires that significantly differ, and for identifying subgroups of repertoires that are most similar to each other compared to the overall population.

The RDI calculation consists of five steps:

- *Step 1: Subsample the repertoire.* When comparing two distinct repertoires, the larger of the two is randomly subsampled to have the same number of elements (reads, molecules, or clones, depending on pre-processing steps) as the smaller repertoire. When multiple repertoires are being compared simultaneously, all repertoires are subsampled without replacement to the size of the smallest repertoire.
- *Step 2: Count abundance of each feature.* Sequences within each repertoire are binned by feature of interest (e.g. V, D, or J gene segments), and the number of elements representing each feature is counted.
- *Step 3: Normalize and transform counts.* In order to improve the consistency of the RDI metric, the total number of clones in each repertoire are normalized to an arbitrary constant (*n* = 500). Optionally, the counts can then be transformed using the ArcSinh function, which is approximately linear for values around zero and logarithmic for values greater than 1.
- *Step 4: Calculate the root mean square deviation of repertoire counts.* Pairwise comparisons of all repertoires are made, and the root mean square deviation (RMSD) (Euclidean distance) between each pair of repertoires is calculated.
- *Step 5: Repeat steps 1–4 and average.* The subsampling process is repeated 100 times, and the RMSD values from all realizations are averaged together to create the final RDI value.

By subsampling all repertoires to the same size, we account for variation due to sequencing depth, thus enabling direct comparison of RDI values regardless of the original repertoire size (Fig. 1b).

One caveat with the subsampling approach is that RDI values will increase as the smallest repertoire size decreases, meaning that the distances only have a defined meaning relative to RDI scores calculated at the same time. Within a set of comparisons, RDI will increase as the differences in repertoire increase, either increasing linearly with the average percent change in gene frequency (if no transformation is used in step 3), or relative to the average log-fold change (if the ArcSinh transformation is used). The latter is recommended for cases where changes in prevalence of less-common genes is of interest, as these changes will otherwise be dominated by the large percentage changes in the most prevalent genes.

### Generation of simulated datasets

To provide a standard reference for the RDI calculation, we used a simulation approach to create datasets with fixed levels of variation. A baseline gene probability vector, **P**_**base**_, was generated containing 50 features with probabilities based on the distribution of gene segments in the IGH, TRA and TRB repertoires from publicly available data [4]. From these baseline vectors, variation was added using a random perturbation vector, **R**, such that:

$$ {\mathbf{P}}_{\mathrm{fc}}={2}^{\left({ \log}_2\left({\mathbf{P}}_{\mathrm{base}}\right)+\mathbf{R}\right)} $$

After perturbation, the resulting probability vector was normalized to sum to 1, and the true deviation of the perturbation vector was calculated, either as the average absolute percent change (for untransformed RDI), or the average absolute log2-fold change (for ArcSinh transformed RDI). The resulting vector was then used to create simulated datasets with known true fold changes.

**Fig. 2:**
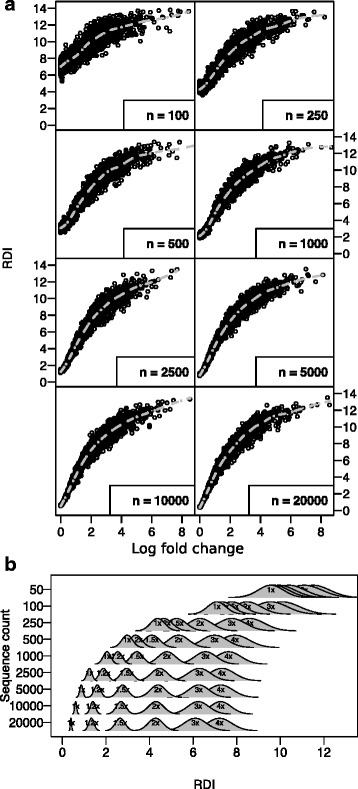
The RDI metric scales with differences in gene frequency. Simulated datasets were generated by randomly drawing genes from a set of fixed probability vectors. Probabilities were generated by perturbing a constant baseline probability vector such that the absolute log-fold difference in each gene was between 0 (no change) and 8 (256-fold increase or decrease in each gene) relative to baseline. Each perturbation vector was used to generate datasets containing varying numbers of sequences (*n* = 50 to 20,000), and a set of equally-sized baseline datasets were generated and compared to the perturbed datasets using the RDI metric. **a** The average RDI score for each perturbed dataset (y axis) is shown against the true average absolute log fold change (relative to baseline) of each perturbation vector (x axis). Spline models were fit to the data (*dotted lines*). **b** Mean and standard deviation of the RDI value was estimated from the spline model at multiple fold change values, and are plotted as probability density functions for a variety of different repertoire sizes (y axis)

Sets of repertoires were generated from each vector by randomly drawing a set number of genes (between 100–10,000) with the given probability. Perturbed repertoires were then compared to repertoires generated from the baseline vector, and the RDI metric was calculated as described above (Fig. 2a). Although the variance in RDI metrics becomes much higher at smaller repertoire sizes, repertoires with 4-fold differences in gene frequency can easily be differentiated with as few as 50 sequences, and with 5000 sequences it’s possible to identify repertoires with extremely small differences. Similar results can be seen if the number of features is changed (Additional file1: Figure S1), where a combination of large repertoire size and large numbers of features results in the highest power to differentiate non-identical repertoires, while a small repertoire size with a large number of features is, conversely, the least powerful.

### Conversion of RDI to fold/percent change values

To account for the RDI metric’s dependence on the input repertoire size, and to make it possible to intuitively understand the magnitude of the differences in two repertoires, we use the simulation approach described above to estimate RDI values for repertoires that vary by set fold changes. For each set of RDI calculations, simulated datasets were generated containing the same number of sequences and genes as the real data. A baseline vector was generated by calculating the average frequency of each gene across the entire dataset, and 2000 distinct **P**_**FC**_ vectors were generated with average fold changes ranging from 1x (no change) to 256x (2^8^ average increase or decrease in each gene). A simulated sequencing dataset was drawn from each perturbed vector, as well as a total of 20 datasets from the baseline vector. For each simulated dataset, the RDI was calculated in comparison to each baseline dataset, and an average RDI was calculated for each initial perturbation vector. This was compared with the true percent change (for untransformed RDI) or fold change (for ArcSinh transformed RDI), which was calculated as the difference between the probability vector and the baseline vector, and a spline model was fit in order to translate from RDI to true difference (Fig. 2a; dotted lines).

### Generation of the RDI ladder

As an alternative to directly converting RDI values to fold/percent change values, local estimates of mean and standard deviation were generated at a set of pre-specified fold/percent change values using the spline model. The calculated spline models were used to estimate the expected RDI value, and the residuals of the model were used to estimate local standard deviation. The mean and standard deviation were then used to generate an approximate distribution of RDI values at a specific fold change (Fig. 2b).

### Datasets

The T cell repertoire data from 5 pairs of identical twins was processed and normalized as previously described [4]. Briefly, naïve and memory T cells were isolated using flow cytometry, and isolated RNA was barcoded and amplified using a RACE based method and sequenced via Illumina MiSeq. Pre-processing of the sequencing data was done using the VDJPipe NGS processing software (manuscript submitted), and consensus sequences were generated for each barcode group using the pRESTO toolkit [16]. For each processed sequence, V, D and J genes and alleles were identified using the IMGT/HighV-QUEST online tool [17]. For the clonally collapsed dataset, sequences were further grouped by clonality using Change-O [8], and each clone was only counted once in all analyses. Shannon entropy was estimated for the V gene frequencies in each repertoire, and the difference in Shannon entropy for naïve vs memory repertoires were calculated within each patient.

## Results

### RDI accounts for heterogeneity in repertoire size and sequencing depth

**Fig. 3:**
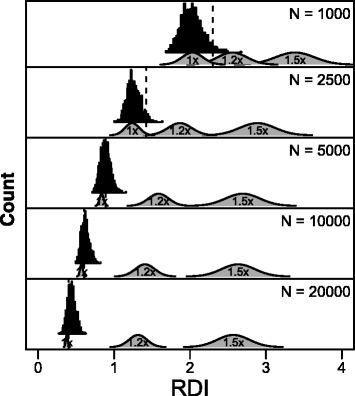
RDI accounts for repertoire size heterogeneity. TRB sequences from a single donor in the Rubelt et al. dataset were randomly assigned to one of two unevenly-sized groups. The smaller group contained 1000, 2500, 5000, 10,000, or 50,000 total sequences, and all remaining sequences were assigned to the second group. V gene frequencies from the two repertoires were compared using the RDI method. The distribution of RDI values across 1000 replicates (black histogram) was compared with simulated data (grey curves) with controlled levels of variance (average fold change of gene segments = 1, 1.2, or 1.5; indicated numbers)

In many cases, it is difficult to differentiate sampling bias during the sequencing process from actual variation in gene prevalence. Using the RDI metric, we determined whether we could identify repertoires that are known to be identical. Clonally collapsed TRB sequences from a single donor were split into two ‘repertoires’, with each clone randomly assigned to one of two unevenly-sized groups. The V gene frequencies within each repertoire were then compared using the RDI metric, and the distribution from 1000 random draws was examined for each repertoire size (Fig. 3). As expected, while the average RDI—as well as the variance of the distance estimate—increase with smaller repertoire sizes, the distribution of RDI values align well with the distribution from simulated datasets.

### T cell repertoire differences are magnified by clonal expansion

**Fig. 4:**
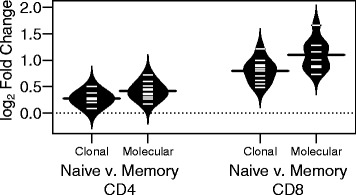
T cell repertoire differences are magnified by clonal expansion. Individual naïve and memory CD4^+^ and CD8^+^ V gene repertoires were tallied based on either the clonally collapsed (clonal) dataset or the full (molecular) dataset from Rubelt et al. Naïve and memory repertoires were then compared within each individual donor (*n* = 10), and log-fold change values were estimated from each RDI value. Individual log-fold change values (*tick marks*) and a kernel density plot (*curved line*) are shown for each group

To characterize the effects of clonal expansion, we chose to compare the V gene frequencies of naïve and memory CD4^+^ and CD8^+^ T cells. Due to the effects of clonal expansion, a subset of T cell clones will be represented by multiple cells within an individual, increasing the prevalence of these expanded clones within the molecular dataset relative to the clonal dataset. In order to examine the effects of this clonal expansion, we characterized V gene usage within each repertoire both in terms of total number of molecules (molecular dataset), or the total number of clones (clonal dataset) containing each gene. RDI values were calculated for each dataset by comparing naïve CD4^+^ or CD8^+^ repertoires with memory repertoires from the same individual, and the RDI values were then converted to fold change values in order to compare across datasets (Fig. 4). Within each cell subset, the fold change values of the molecular repertoires were significantly higher than the clonal repertoires (paired *t*-test; CD4^+^: *p* < 0.001; CD8^+^: *p* < 0.001), reflecting the added variation resulting from clonal expansion. In addition, the fold changes in the CD8^+^ repertoire were significantly higher than the CD4^+^ fold changes, both in the clonal dataset (paired *t*-test *p* < 0.001), and in the molecular dataset (*p* < 0.001). Finally, the difference between the clonal repertoires and the molecular repertoires—i.e. the effect of clonal expansion on gene prevalence—was also slightly, but significantly, higher in the CD8^+^ repertoire than in CD4^+^, with fold-increases in dissimilarity of 1.2-fold in the CD8^+^ repertoire compared with 1.1-fold in the CD4^+^ repertoire (*p* < 0.001). Taken together, these findings suggest that clonal expansion introduces biases into the repertoires of both CD4^+^ and CD8^+^ repertoires, but that these biases are larger in the CD8^+^ repertoire than in the CD4^+^ repertoire.

Given that the changes in the memory repertoire are most likely due to increased clonal expansion, it is likely that the changes in composition, as measured by RDI, will be associated with equivalent changes in the diversity of the repertoire. In order to measure this, we characterize the diversity within each naïve and memory repertoire using the Shannon index, and calculated a fold change for each sample. As expected, the fold changes calculated by RDI were well-correlated with the fold changes in Shannon entropy scores (Additional file 2: Figure S2a). However, the changes in the CD8^+^ diversity were not significantly greater than the changes in CD4^+^ diversity for the same patients, although the trend remained consistent (paired *t*-test *p* = 0.053; Additional file 2: Figure S2b).

## Discussion

Direct comparison and quantification of genetic repertoires is a difficult problem, complicated by the high variance in prevalence of gene segments coupled with inconsistencies in sequencing depth between repertoires. In this paper, we present a novel method for quantifying repertoire gene abundance differences: the Repertoire Dissimilarity Index (RDI). This method uses a non-parametric subsampling approach to account for variance in repertoire size, and, coupled with a data simulation approach, allows for direct quantification of the average variation between repertoires.

In this paper, we demonstrate that the RDI metric accurately accounts for the variance-inflating effects of low sequencing depth in a straightforward and easy-to-understand way. Furthermore, using simulated datasets, we show that these distance metrics are directly proportional to the average fold change in gene prevalence, independent of total repertoire size. The ability to meaningfully quantify the variation between repertoires is important, as it enables an intuitive understanding of the differences between a pair of repertoires. Additionally, the conversion of RDI values to standard units—in this case either log fold change or percent change—allows for comparison across datasets. We demonstrate the utility of this conversion here by calculating RDIs within a molecular dataset and a clonal dataset separately, and then comparing the results.

An important feature of the RDI metric is that it is agnostic to the specific types or numbers of features being considered. We demonstrate that RDI can detect meaningful biological differences when used with anywhere between 5 to 1,000 features, and it is likely that RDI will be equally useful in analyses of specific V(D)J rearrangements (which contain as many as 10,000 potential features). However, while there doesn’t appear to be any point at which the RDI estimates are incorrect, the variance of the metric can be quite high in experiments with low depth and a large numbers of features (see Additional file 1: Figure S1). A handy rule of thumb for these experiments appears to be that, in order to detect differences of 2-fold or greater with reasonable power, the number of sequences must be equal to or greater than the number of features.

The RDI has much in common with other repertoire diversity metrics, such as Shannon entropy, species richness, and Hill diversity. Both types of metrics can be used to study the effects of clonal expansion within a repertoire, and both can be used to quantify the differences between a naïve and memory repertoire within an individual. However, diversity metrics are not designed to take into account the contents of a repertoire, and repertoires with equivalent levels of clonal expansion may still have the same overall diversity despite having entirely different contents. Within an individual, the differences between the content of, e.g., naïve and memory repertoires are less drastic, and we saw that the change in diversity correlates well with the differences in content. However, this will most likely not be the case for comparisons between individuals, where differences in content will be the major factor affecting the RDI metric.

The Repertoire Dissimilarity Index was developed to aid in the direct comparison of repertoires in a set of identical twins [4]. In Rubelt et al., the RDI was used to identify striking differences in heritability of various immune compartments. Using direct comparisons of B and T cell repertoires, among others we confirmed previous reports showing a MZ twin bias in the choice of V-J combinations in naïve B and T cells, and extended this finding into the memory repertoire.

In this paper, we provide a follow-up to the analyses in Rubelt et al., and examine the effects of clonal expansion among the memory compartments of CD4^+^ and CD8^+^ T cells. Clonal expansion is the process by which antigen-specific B or T cell becomes activated and rapidly divides over multiple generations, and is an important step in the response to specific pathogens. As expected, we see that clonal expansion acts to increase the average variability in the memory repertoire, implying that clones are expanded in a targeted way, irrespective of their prevalence among naïve cells. Although this is a well-known mechanism, the differences between the CD4^+^ and CD8^+^ repertoires are less expected. Our results show that, compared to CD4^+^, more variation is introduced into the CD8^+^ memory repertoire during the antigen-driven selection process, and that clonal expansion further increases these differences by introducing more variation into the CD8^+^ repertoire. This is consistent with previous reports that CD8^+^ repertoires are subject to higher levels of clonal expansion, with greater proliferation of activated clones compared to CD4^+^ repertoires [18].

While the differences in the molecular dataset can be primarily explained by differences in clonal expansion, the difference between CD4^+^ and CD8^+^ within the clonal collapsed dataset implies that CD4^+^ and CD8^+^ also vary in terms of clonal selection; i.e. the process by which a naïve T cell clone is exposed to antigen and transitions to the activated/memory T cell pool. When the naïve CD8^+^ repertoire was compared with the memory repertoire, we observed significantly higher levels of variation compared to the CD4^+^ repertoire. This implies that the CD4^+^ repertoire selects and expands a wider variety of clones from among the naïve repertoire, whereas CD8^+^ is more selective in which clones respond to antigens and subsequently transition to the memory compartment. This is further supported by observations by [4], where the memory CD8^+^ repertoires of identical twins were less similar than those twins’ CD4^+^ memory repertoires. In addition, striking examples of the increased specificity of the CD8^+^ repertoire can be observed in responses to latent cytomegalovirus infection, where specific CD8^+^ cells often represent 10-20% of all CD8^+^ cells by peptide-MHC tetramer analysis, whereas CD4^+^ responses are more diverse and rarely comprise more than 1% of CD4^+^ cells [19–22]. Taken together, these results suggest that CD8^+^ T cell clones have to undergo a stricter selection process compared to CD4^+^, and that this process will result in a more specialized CD8^+^ memory repertoire within each individual.

Although greater selective bias in the CD8^+^ repertoire is most likely the primary cause of the increased variance, several other factors may contribute to this increased variance. One possible source of variation is the presence of terminally differentiated T cells, a relatively uncommon subset of CD8^+^ T cells that are CD45RO^−^ CCR7^−^, and were thus among the cells isolated in the naïve T cell population [23]. Although we only expect them to make up a relatively small portion of the naïve CD8^+^ T cell population, these terminally differentiated T cells will have a unique repertoire of receptors, thus increasing the variance when compared to the memory repertoire. Additionally, although the clonal dataset has been collapsed such that only one sequence from each clonal group was counted, the most expanded clones will still have a higher likelihood of recovery compared to the non-expanded clones, resulting in a slight but measurable skewing of the CD8^+^ repertoire towards these expanded clones. Both of these factors would most likely have a slight, but non-zero, variance-inflating effect on the CD8^+^ memory repertoire.

## Conclusions

Immune repertoire profiling is still a relatively new field of research, and accepted tools for analysis of repertoires are still lacking. In this paper we present the Repertoire Dissimilarity Index, a powerful and easy-to-interpret metric for the comparison of immune repertoires. This tool will be useful for all analyses of immune sequences, and can easily be extended for use in any repertoire experiment.

## Additional files

Additional file 1: Figure S1. RDI values vary according to the number of genes. Simulated datasets were generated by randomly drawing genes from a set of fixed probability vectors. Probabilities were generated by perturbing a constant baseline probability vector such that the absolute log-fold difference in each gene was between 0 (no change) and 8 (256-fold increase or decrease in each gene) relative to baseline. Each perturbation vector was used to generate datasets containing varying numbers of sequences (*n* = 50 to 20,000), and were then compared against a set of baseline datasets containing the same number of sequences. Mean and standard deviation of the RDI value was estimated from the spline model at multiple fold change values, and are plotted as probability density functions for a variety of different repertoire sizes (y axis). (PDF 59 kb)
Additional file 2: Figure S2. Changes in repertoire content correlate with diversity changes following clonal expansion. Individual naïve and memory CD4^+^ and CD8^+^ V gene repertoires were tallied based on the full (molecular) dataset from Rubelt et al. Shannon entropy was calculated for each repertoire, and the fold change in entropy between the naïve and memory repertoires of each patient/cell type. A) Absolute log_2_ fold change values of Shannon entropy are plotted against the estimated fold change in repertoire contents as calculated by RDI. B) Individual log-fold change values (tick marks) and a kernel density plot (curved line) are shown for each group. Significance was determined using a paired *t*-test. (PDF 16 kb)

## Abbreviations

Ig: Immunoglobulin
IGH: Immunoglobulin heavy domain
RACE: Rapid amplification of cDNA ends
RDI: Repertoire dissimilarity index
RMSD: Root mean square deviation
TCR: T cell receptor TRA – T cell receptor, Alpha chain
TRB: T cell receptor, Beta chain
