# Supplementary figures and images for: The Repertoire Dissimilarity Index as a method to compare lymphocyte receptor repertoires
Source: BMC Bioinformatics. 2017 Mar 7;18:155. doi: 10.1186/s12859-017-1556-5 (PMC5340033; doi:10.1186/s12859-017-1556-5)

**Genes = 5**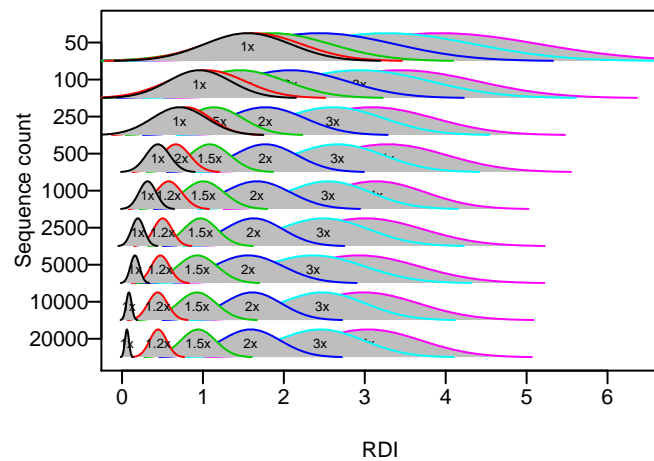**Genes = 25**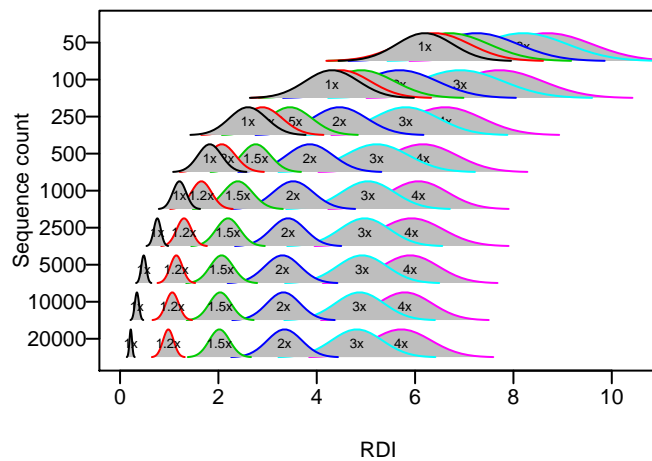**Genes = 50**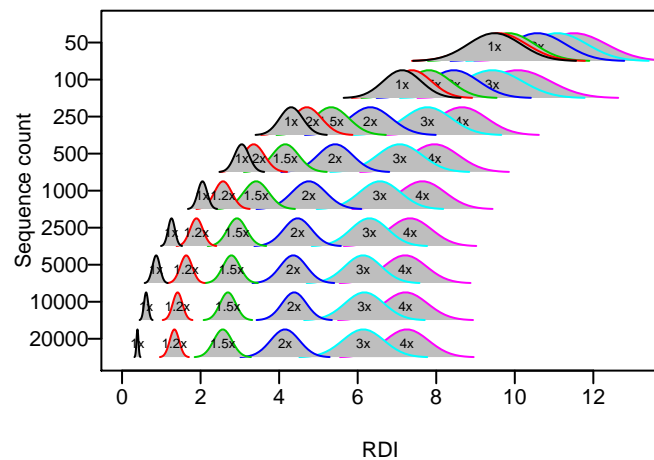**Genes = 100**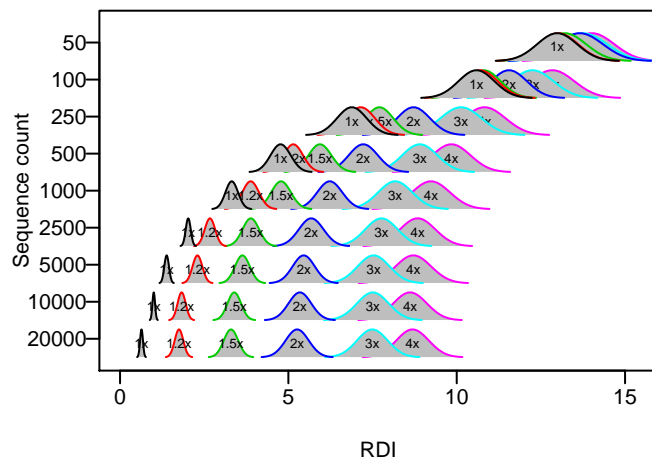**Genes = 500**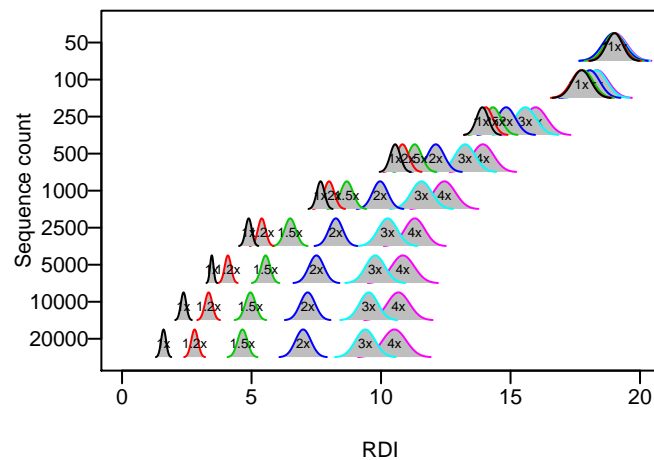**Genes = 1000**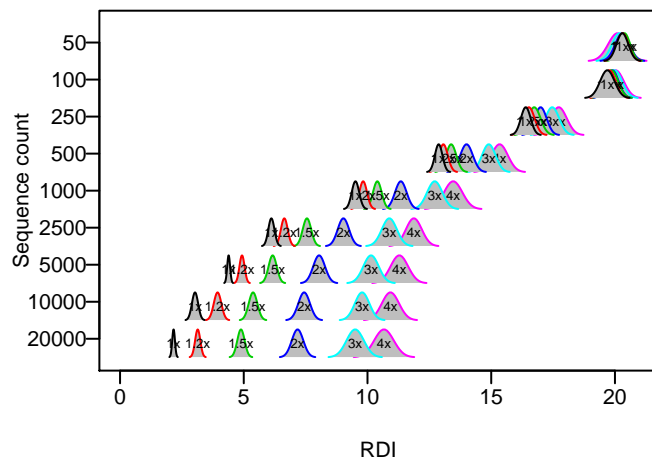

Supplement: Additional file 1: Figure S1. — RDI values vary according to the number of genes. Simulated datasets were generated by randomly drawing genes from a set of fixed probability vectors. Probabilities were generated by perturbing a constant baseline probability vector such that the absolute log-fold difference in each gene was between 0 (no change) and 8 (256-fold increase or decrease in each gene) relative to baseline. Each perturbation vector was used to generate datasets containing varying numbers of sequences (n = 50 to 20,000), and were then compared against a set of baseline datasets containing the same number of sequences. Mean and standard deviation of the RDI value was estimated from the spline model at multiple fold change values, and are plotted as probability density functions for a variety of different repertoire sizes (y axis). (PDF 59 kb) [file 12859_2017_1556_MOESM1_ESM.pdf]

**CD4**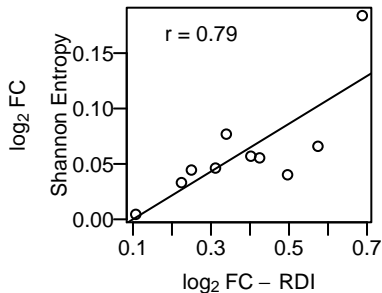**CD8**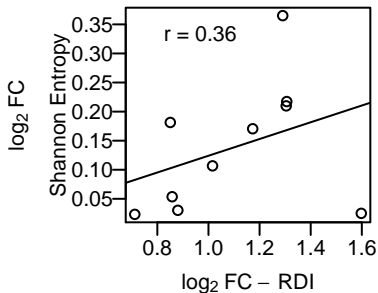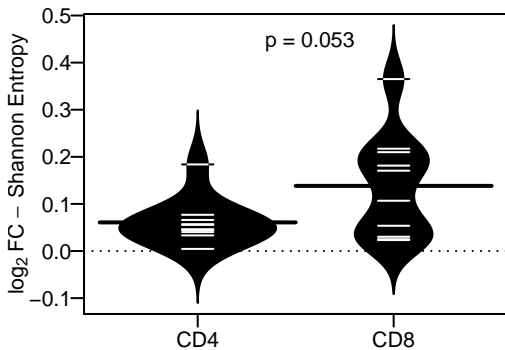

Supplement: Additional file 2: Figure S2. — Changes in repertoire content correlate with diversity changes following clonal expansion. Individual naïve and memory CD4+ and CD8+ V gene repertoires were tallied based on the full (molecular) dataset from Rubelt et al. Shannon entropy was calculated for each repertoire, and the fold change in entropy between the naïve and memory repertoires of each patient/cell type. A) Absolute log2 fold change values of Shannon entropy are plotted against the estimated fold change in repertoire contents as calculated by RDI. B) Individual log-fold change values (tick marks) and a kernel density plot (curved line) are shown for each group. Significance was determined using a paired t-test. (PDF 16 kb) [file 12859_2017_1556_MOESM2_ESM.pdf]
